# SARS-CoV-2 infection and dysregulation of nuclear factor erythroid-2-related factor 2 (Nrf2) pathway

**DOI:** 10.1007/s12192-023-01379-0

**Published:** 2023-10-05

**Authors:** Rabab S. Hamad, Hayder M. Al-kuraishy, Athanasios Alexiou, Marios Papadakis, Eman A. Ahmed, Hebatallah M. Saad, Gaber El-Saber Batiha

**Affiliations:** 1 Biological Sciences Department, College of Science, King Faisal University, 31982 Al Ahsa, Saudi Arabia; 2 Central Laboratory, Theodor Bilharz Research Institute, Giza, 12411 Egypt; 3 Department of Pharmacology, Toxicology and Medicine, Medical Faculty, College of Medicine, Al-Mustansiriyah University, P.O. Box 14132, Baghdad, Iraq; 4 Department of Science and Engineering, Novel Global Community Educational Foundation, Hebersham, NSW 2770 Australia; 5 AFNP Med, 1030 Vienna, Austria; 6 Department of Surgery II, University Hospital Witten-Herdecke, University of Witten-Herdecke, Heusnerstrasse 40, 42283 Wuppertal, Germany; 7 Department of Pharmacology, Faculty of Veterinary Medicine, Suez Canal University, Ismailia, 41522 Egypt; 8 Department of Pathology, Faculty of Veterinary Medicine, Matrouh University, Marsa Matruh, 51744 Egypt; 9 Department of Pharmacology and Therapeutics, Faculty of Veterinary Medicine, Damanhour University, Damanhour, 22511 Egypt

**Keywords:** COVID-19, Nuclear factor erythroid 2-related factor 2, Oxidative stress, SARS-CoV-2

## Abstract

Coronavirus disease 2019 (COVID-19) is a recent pandemic caused by a novel severe acute respiratory syndrome coronavirus 2 (SARS‑CoV‑2) leading to pulmonary and extra-pulmonary manifestations due to the development of oxidative stress (OS) and hyperinflammation. The underlying cause for OS and hyperinflammation in COVID-19 may be related to the inhibition of nuclear factor erythroid 2-related factor 2 (Nrf2), a master regulator of antioxidative responses and cellular homeostasis. The Nrf2 pathway inhibits the expression of pro-inflammatory cytokines and the development of cytokine storm and OS in COVID-19. Nrf2 activators can attenuate endothelial dysfunction (ED), renin-angiotensin system (RAS) dysregulation, immune thrombosis, and coagulopathy. Hence, this review aimed to reveal the potential role of the Nrf2 pathway and its activators in the management of COVID-19. As well, we tried to revise the mechanistic role of the Nrf2 pathway in COVID-19.

## Introduction

The whole world confronted a disaster situation that first emerged in late December 2019 as purely a few cases of pneumonia in Wuhan, China (Batiha et al. 2021a). A scrupulous investigation employing next-generation sequencing and phylogenetic analysis led to the recognition of the causative agent of this respiratory disease, a novel coronavirus (2019-nCoV) (Al-Kuraishy et al. 2021a). The World Health Organization (WHO) allocated a name,Coronavirus disease 2019, or COVID-19, to the disease and declared it a pandemic on March 11, 2020 (McFee 2020). Later on, the 2019-nCoV was renamed to SARS-CoV-2 by the International Committee on Taxonomy of Viruses based on its genetic match to a previously known coronavirus, severe acute respiratory syndrome coronavirus (SARS-CoV) (McFee 2020). Transmission of SARS-CoV-2 occurs when a healthy individual inhales or comes into contact with respiratory droplets from an infected person (Al-Kuraishy et al. 2022n). The average incubation period before a patient exhibits disease symptoms ranges from 2 to 14 days (El-Saber Batiha et al. 2022). SARS-CoV-2 has shown that it is genetically similar to previously known coronavirus SARS-CoV and hence is placed under the family *Coronaviridae* (Al-Kuraishy et al. 2021k). Coronavirus contains positive-sense single-stranded RNA as its genetic material which also helps the virus to evade host immune response and assists its entry inside the host cell (Al-Kuraishy et al. 2021l). Interestingly, SARS-CoV-2, similar to SARS-CoV, exploits the angiotensin-converting enzyme 2 (ACE2) receptor to gain access inside human cells (Babalghith et al. 2022). Besides, the trimeric S protein of SARS-CoV-2 is sliced by transmembrane protease serine 2 (TMPRSS2), similar to SARS-CoV (Al-Kuraishy et al. 2021c).

**Fig. 1:**
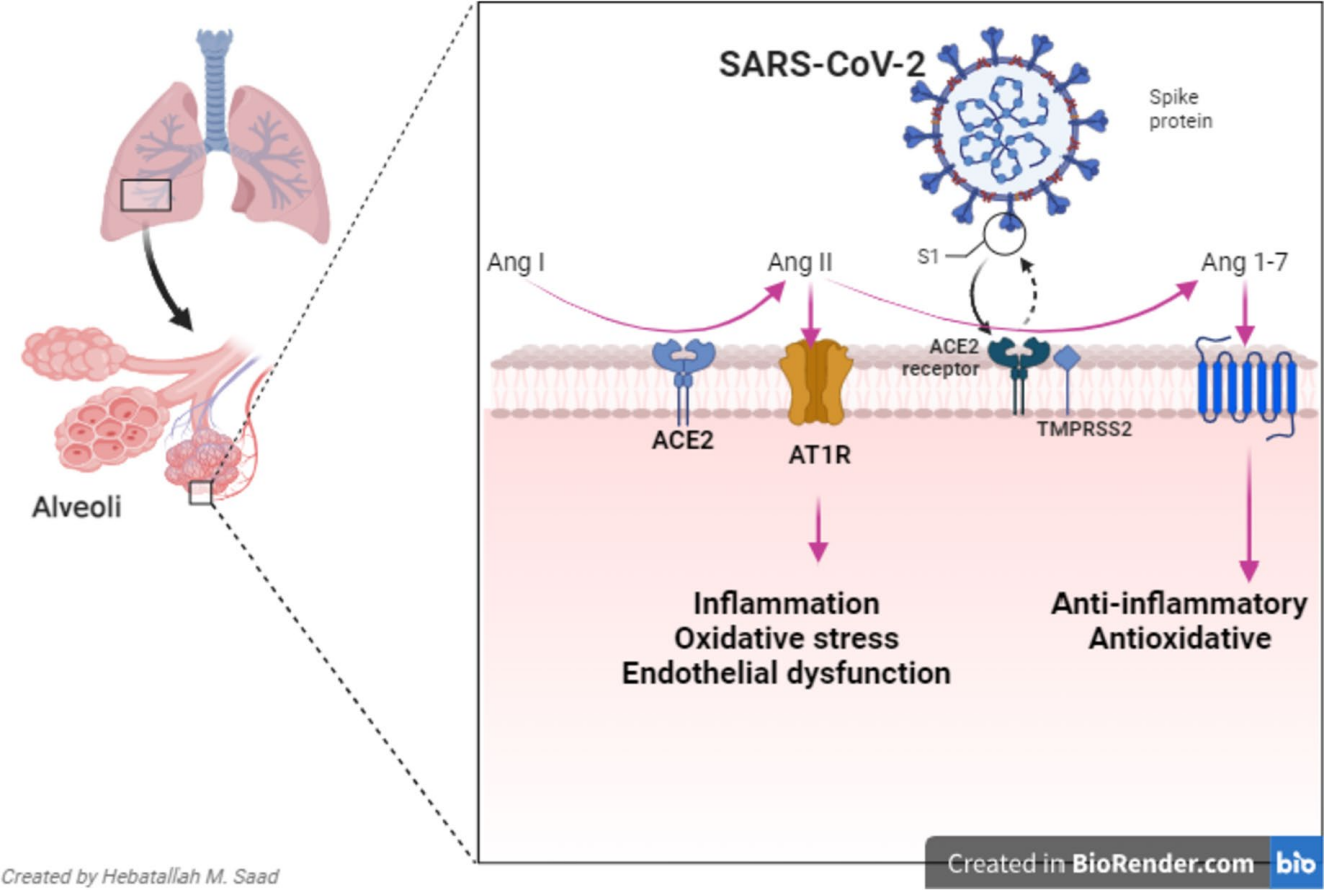
Pathophysiology of SARS-CoV-2 infection

The peptidase ACE2 metabolizes vasoconstrictor angiotensin II (Ang II) to the vasodilator Ang1-7 and Ang1-9 (Al-Kuraishy and Al-Gareeb 2020). ACE2 receptor is highly expressed in various cellular systems, including enterocytes, cardiomyocytes, pulmonary alveolar cells, neurons, and testes (Moubarak et al. 2021). Consequently, the downregulation of ACE2 during SARS-CoV-2 infection provokes vasoconstriction and the development of endothelial dysfunction (ED), oxidative stress (OS), and inflammatory disorders (Al-Kuraishy et al. 2022g). The binding of SARS-CoV-2 with the ACE2 receptor leads to a series of inflammatory cellular events with cytopathic effects causing cell injury and hyperinflammation (Al-Thomali et al. 2022a) (Fig. 1).

The clinical presentation of COVID-19 is mainly asymptomatic or presented with mild symptoms in 85% of cases (Al-Kuraishy et al. 2022y). Nonetheless, 15% presented with moderate-severe form due to the progress of acute lung injury (ALI) (Mostafa-Hedeab et al. 2022a). Also, 5% of COVID-19 patients may be critical and necessitate assessed ventilation due to the development of acute respiratory distress syndrome (ARDS) (Al-Kuraishy et al. 2022p).

SARS-CoV-2-induced OS triggers activation of different signaling pathways such as nuclear factor erythroid 2-related factor 2 (Nrf2), which induces cellular interactions to mitigate SARS-CoV-2-mediated viral toxicity and cellular injury (Zhu et al. 2021). The Nrf2 is a transcription factor that normalizes numerous essential genes that encode body antioxidant and anti-inflammatory signaling systems (Zhu et al. 2021). Downregulation of Nrf2 by SARS-CoV-2 is associated with an unregulated expression of the ACE2 receptor and the development of OS and inflammatory disorders (Qu et al. 2023). Consequently, the objective of the present review was to elucidate the role of Nrf2 in SARS-CoV-2 infection.

## Overview of the Nrf2

Nrf2, a master regulator of antioxidative responses, is very crucial in maintaining cellular homeostasis (Vomund et al. 2017). Nrf2 belongs to the NFE2 family of transcription factors and contains seven Neh domains that regulate Nrf2 activity by binding to DNA or proteins (Vomund et al. 2017). Nrf2 is a transcription factor that regulates the expression of antioxidant enzymes and antioxidant response elements during the development of OS and inflammatory reaction (Pall and Levine 2015). Nrf2 is activated in two ways; in canonical Nrf2 activation, specific cysteine residues on Keap1 are oxidized by oxidative stress or electrophiles, resulting in a conformational change in the adaptor protein and the inhibition of E3 ubiquitin ligase activity (Gan et al. 2010). Alternatively, non-conical mechanisms can disturb the interaction of keap1 and Nrf2 through Nrf2 phosphorylation (Gan et al. 2010).

**Fig. 2:**
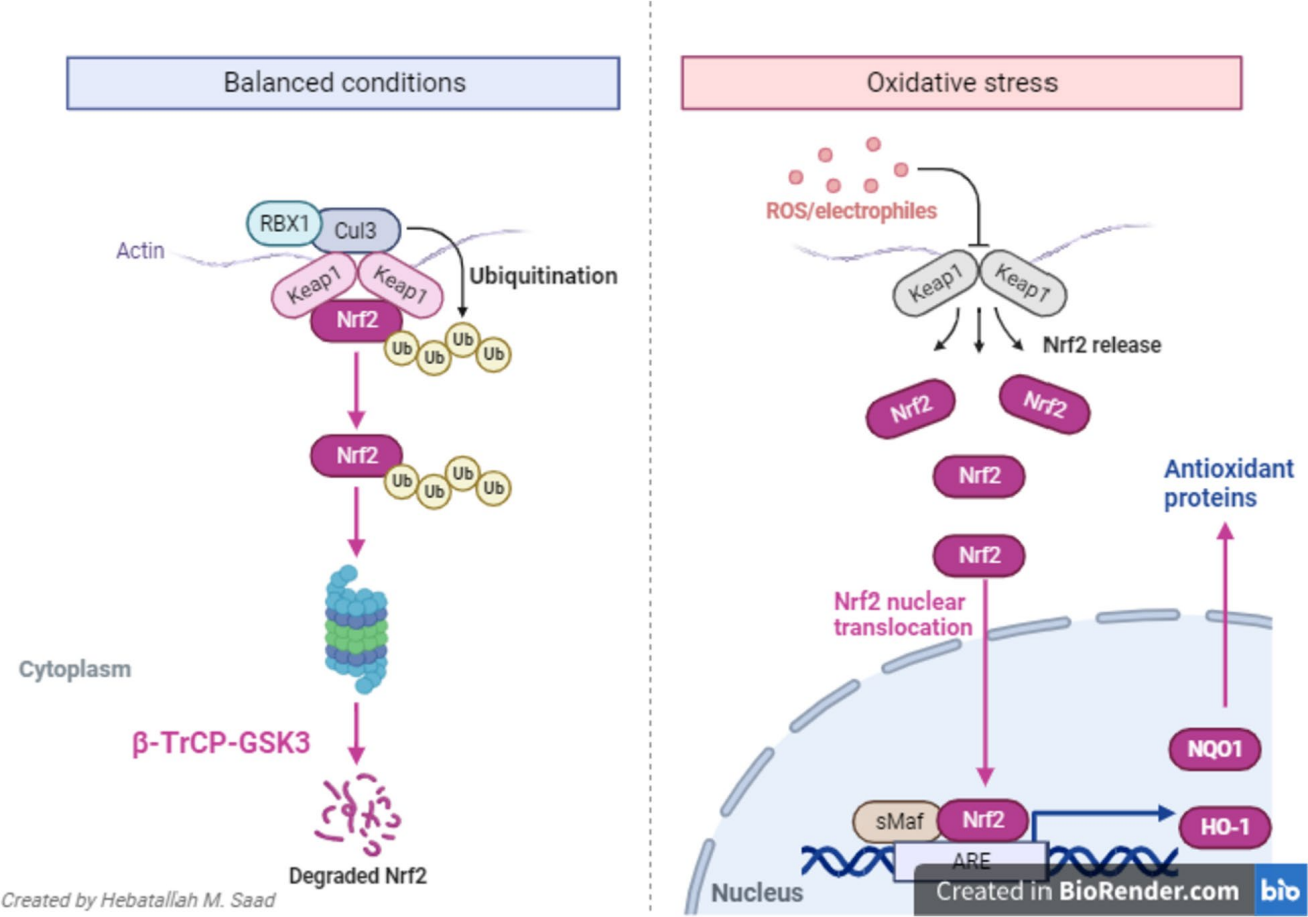
Molecular mechanism of nuclear factor erythroid 2-related factor 2 (Nrf2): (1): Under balanced conditions, Nrf2 is anchored with Kelch-like ECH-associated protein 1 (KEAP1). Nrf2 binds keap, gets ubiquinat3d, and degraded by β-TrCP in the cytoplasm. (2): Under oxidative stress, Nrf2 is dissociated and enters and binds small maf protein (sMaf) to bind antioxidant response element (ARE), which increases the expression of the antioxidant gene. Nrf2 is degraded by beta-transducin repeat-containing protein glycogen synthase kinase 3 (β-TrCP-GSK3). Dissociation of Nrf2 from Kelch-like E.C.H. associated protein 1 (KEAP1) with activation of antioxidant response element (ARE), which increases the expression of antioxidant genes heme oxygenase 1 (HO-1) and quinone oxidoreductase (NQO1), which blocks the progression of oxidative stress (OS) and maintains redox balance and cytoprotective effect

Nrf2 triggers the expression of phase II enzymes and heme oxygenase 1 (HO-1), and inhibiting inflammatory signaling pathways (Gan et al. 2010). As well, Nrf2 has pleiotropic effects in controlling the immune response, and cellular metabolism (Jung and Kwak 2010). Noteworthy, Nrf2 is engaged with Kelch-like ECH-associated protein 1 (Keap1), which regulates the anti-inflammatory and antioxidant effects of Nrf2 (Zhong et al. 2019). Keap1 regulates the expression of adaptor protein and ubiquitin ligase complex via binding Culin-3 and Rbx1 with an anchoring effect on the cytoplasmic Nrf2 (Hikichi et al. 2019). During the development of OS and generation of reactive oxygen species (ROS), Nrf2 is rapidly dissociated from KEAP1 and translocated to the nucleus and activation the expression of antioxidant proteins to maintain cellular homeostasis (Li et al. 2014). Following the ending of OS, Nrf2 is inactivated by cytoplasmic KEAP1 and nuclear beta-transducing repeat-containing protein glycogen synthase kinase 3 (β-TrCP-GSK3) (Iizuka et al. 2021) (Fig. 2).

Furthermore, Nrf2 supports different metabolic processes such as the production of nicotinamide adenine dinucleotide phosphate (NADPH) and the metabolism of amino acids, lipids, nucleotides, and iron/heme (DeBlasi and DeNicola 2020). Nrf2 activates glutaminase, which converts glutamine into glutamate which enter the nucleus and involved in the production of GSH (Hamada et al. 2021). Nrf2 significantly influences the regulation of the enzymes involved in the production pathway of serine which is a component of different macromolecules, such as nucleotides, ceramide, and sphingolipid (Ishii et al. 2022). As well, Nrf2 is a critical factor in cellular components, which helps in cellular repair and OS control (Yu and Xiao 2021).

Of interest, Nrf2 attenuates OS-induced ALI/ARDS by mitigating endothelial dysfunction. In an in vitro study conducted, Canella et al. (Canella et al. 2018) illustrated that Nrf2 pathway activators prevent OS-induced ALI via mitigation of outward rectifier chloride channels (ORCCs) in human lung epithelial cells (A549 line). On the other hand, Wu and colleagues revealed that the Nrf2 pathway attenuates the development of diabetic cardiomyopathy through the mitigation of OS (Wu et al. 2022). Flavonoids have been shown to mitigate OS in cell lines via activation of the Nrf2 pathway (Sindhu et al. 2021). Similarly, chrysin reduces sepsis-induced cardiac injury by ameliorating the Nrf2 pathway (Xingyue et al. 2021). Furthermore, the underlying mechanism of the antioxidant effects of Nrf2 is through the induction expression of antioxidant protein genes like HO-1 and quinone oxidoreductase (NQO1), which block the progression of OS and maintain redox balance (Sugimoto et al. 2021). These findings suggest that Nrf2 reduces the propagation of OS-induced tissue injury and organ dysfunction.

## Nrf2 and SARS-CoV-2 infection

In SARS-CoV-2 infection, Nrf2 is highly dysregulated, causing abnormal expression of ACE2 with further increasing viral entry (Nguyen et al. 2022). SARS-CoV-2 induces protein kinase receptor (PKR) activation, which acts as endoplasmic reticulum kinase to promote the degradation of Nrf2 (Mostafa-Hedeab et al. 2022b). The Nrf2 pathway is inhibited during SARS-CoV-2 infection leading to augmentation of OS and related inflammatory disorders (Nguyen et al. 2022). Therefore, NF-κB and NADPH oxidases are activated in SARS-CoV-2 infection, causing hyperinflammation and OS, respectively (Al-Kuraishy et al. 2022q). In addition, Nrf2 inhibits the activation of stimulator of interferon genes (STING) which regulate the expression of interferon (IFN) response (Ryan et al. 2022). In turn, IFN blocks the expression of Nrf2, leading to hyperinflammation and OS (Ryan et al. 2022). Concerning the clinical significance of Nrf2 level in COVID-19 patients, a comparative study including 40 children with COVID-19 compared with matched 35 healthy controls showed that Nrf2 level was lower in children with COVID-19 as compared with healthy controls due to tissue damage and OS (Gümüş et al. 2022). Furthermore, various studies suggested that Nrf2 level is highly dysregulated in COVID-19 patients (Cuadrado et al. 2020; Singh et al. 2021).These findings proposed that exaggerated immune response in SARS-CoV-2 infection induces a substantial reduction in the activity of the Nrf2 pathway.

Moreover, Nrf2 improves antiviral response against different viral infections for example Nrf2 activators restrict the replication of herpes simplex virus-1 (HSV-1) in human primary fibroblasts (Wyler et al. 2019). Likewise, Nrf2 protects against infections with the respiratory syncytial virus (RSV) and metapneumovirus (MNV) by modifying the innate immune response and preventing viral replication. Infections with RSV and MNV are associated with OS formation and hyperinflammation, which promote Nrf2 gene expression (Ivanciuc et al. 2018). Additionally, it has been noted that Nrf2 is essential for preventing OS-induced neurocognitive problems in people with human immunodeficiency virus (HIV) (Reddy et al. 2012). Reddy et al. (Reddy et al. 2012) found that HIV glycoprotein 120 induces expression of the Nrf2/HO-1/NQO1 axis in parallel with the activation of pro-inflammatory cytokines, mainly TNF-α (Reddy et al. 2012). Furthermore, Nrf2 is upregulated in response to the chronic effects of viral infections. In addition, Nrf2 prevents the spread of the influenza virus infection (Ramezani et al. 2018). Nrf2 is a major regulator during viral infections; some infections activate Nrf2, though other viral infections may provoke Nrf2 independent of the antioxidant pathway (Cherupanakkal et al. 2017). Remarkably, Nrf2 is involved in the host immune response in patients with dengue infection (Cherupanakkal et al. 2017). According to comparison research involving 88 dengue patients and 31 patients with other febrile illnesses, dengue patients had higher levels of Nrf2 expression in their human peripheral mononuclear cells than those with other febrile illnesses (Cherupanakkal et al. 2017). Therefore, Nrf2 may be implicated in the virulence and pathogenesis of viral infections. These findings proposed that Nrf2 plays a crucial role in viral infections to counteract the associated OS and exaggerated inflammatory reactions. Nevertheless, Nrf2 could be implicated in the propagation of viral infections.

**Fig. 3:**
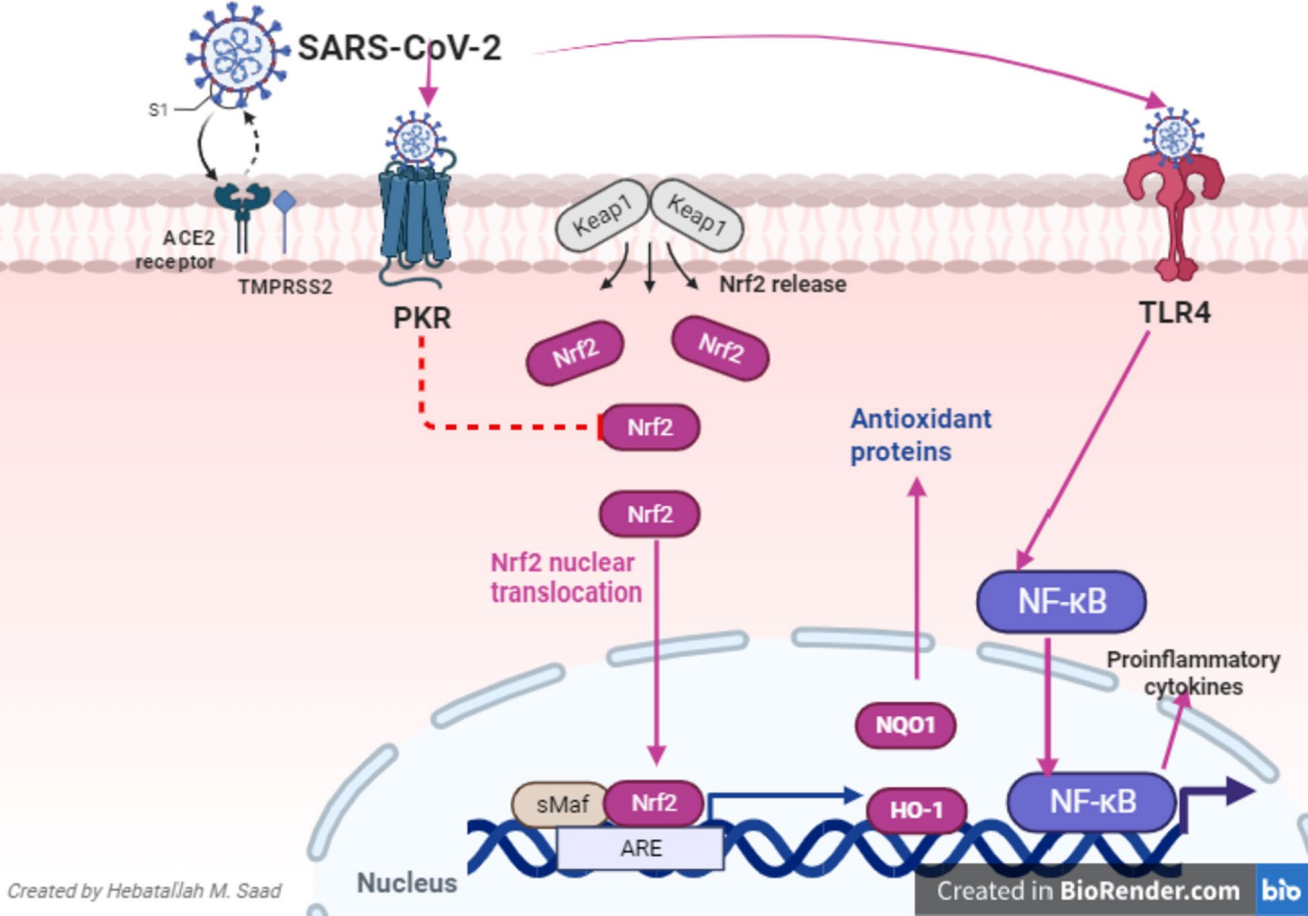
Nuclear factor erythroid 2-related factor 2 (Nrf2) and SARS-CoV-2: SARS-CoV-2 induces protein kinase receptor (PKR) activation that promote the degradation of NRF2. Toll-like receptor (TLR) signaling promotes the expression of nuclear factor kappa B (NF-κB), which plays an essential role in primary immune response and induction of inflammation. Nrf2 has direct anti-inflammatory by inhibiting the NF-κB-dependent release of pro-inflammatory cytokines. Nrf2 produces an anti-inflammatory effect through the activation of heme oxygenase 1 (HO-1)

Nrf2 has an integral role in the regulation of immune response and propagation of inflammation (Vomund et al. 2017). It reduces the expression of pro-inflammatory cytokines, including MCP-1, TNF-α, IL-6, and IL-1β, by inhibiting the recruitment of RNA polymerase II and macrophage activation (Battino et al. 2018). Interestingly, Nrf2 blocks the expression of NF-κB, which plays an essential role in primary immune response and induction of inflammation (Lee et al. 2015). Therefore, depletion of Nrf2 signaling enhances lipopolysaccharide (LPS)-induced lung inflammation by exaggerating NF-κB/TNF-α (Rushworth et al. 2011). Yan and coworkers revealed that Nrf2 signaling attenuates ALI and lung inflammation development in mice by inhibiting the expression of TLR4 (Yan et al. 2018). The net effect of Nrf2 on the inflammatory reaction is through the activation of HO-1, which produces an anti-inflammatory effect. As well, Nrf2 has a direct anti-inflammatory by inhibiting the NF-κB-dependent release of pro-inflammatory cytokines (Fig. 3).

## Mechanistic role of Nrf2 in SARS-CoV-2 infection

### Nrf2 and inflammatory signaling pathways

#### NLRP3 inflammasome

Nod-like receptor family, pyrin domain-containing 3 (NLRP3) inflammasome are multiprotein complexes formed in the cytosol driving caspase-1 cleavage and the secretion of the pro-inflammatory cytokines IL-1β and IL-18 and other damage-associated molecular patterns (DAMPs) (Batiha et al. 2022a; Alrouji et al. 2023a). NLRP3 inflammasome promotes antigen presentation and the induction of an adaptive immune response (Batiha et al. 2023a).

It has been shown that SARS-CoV-2 infection and abnormal immune response are associated with the activation of different inflammatory signaling pathways leading to hyperinflammation (Al-Kuraishy et al. 2022f). In COVID-19, an excessive immunological response results in a high level of NLRP3 inflammasome activation (Al-Kuraishy et al. 2021j). In the SARS-CoV-2 infection, the endogenous adjuvant activity is caused by the direct activation of NLRP3 by a viral protein, named viroporin protein 3a, suggesting that SARS-CoV-2 can directly activate NLRP3 inflammasome (Al-Kuraishy et al. 2022t). Targeting of NLRP3 inflammasome pathway by selective inhibitors may reduce COVID-19-induced complications (Batiha et al. 2021b). Liu et al. (Liu et al. 2017a) showed that Nrf2 negatively regulates the expression of NLRP3 inflammasome via inhibition of ROS generation (Liu et al. 2017b). An experimental study demonstrated that rotenone-induced NLRP3 inflammasome expression by ROS was inhibited by Nrf2 mediated activation of NQO1 in mice (Liu et al. 2017b). Therefore, by inhibiting the NLRP3 inflammasome in COVID-19, Nrf2 activators may significantly decrease SARS-CoV-2 infection-induced inflammation (Mendonca and Soliman 2020). Besides, activation of NLRP3 inflammasome is associated with more expression of NF-κB, which increases inflammatory disorders via the release of pro-inflammatory cytokines in COVID-19 (Batiha et al. 2022b). Importantly, Nrf2 attenuates the expression of NF-κB, leading to potent anti-inflammatory effects (Mendonca and Soliman 2020). It has been observed that the expression of NLRP3 was upregulated, and the expression of IL-1β and IL-18 was downregulated after Nrf2 silencing (Chen et al. 2019). It has been reported that bardoxolone methyl offers an effective pharmacological approach to increasing Nrf2 activity and mitigating cholestasis in hepatic ischemia–reperfusion injury (Ruiz et al. 2013). Nrf2 activator isoliquiritigenin prevents the development of ALI in mice by suppressing the NF-κB pathway through induction expression of Nrf2 and adenosine monophosphate protein kinase (AMPK) (Liu et al. 2017a).

##### TLR4 and high mobility box protein 1 (HMBP1)

Interestingly, immune response during acute cell injury promotes the expression of TLR4 and HMBP1 (Alkhayyat et al. 2022). Nrf2 reduces the expression of pro-inflammatory cytokines, including MCP-1, TNF-α, IL-6 and IL-1β by inhibiting the recruitment of RNA polymerase II and macrophage activation (Yu et al. 2019).Therefore, Nrf2 attenuates the development of inflammatory changes straight by its anti-inflammatory effects or indirectly via modulation the expression of HO-1 and reduction of OS (Saha et al. 2020). In COVID-19, the exaggerated expression of TLR4 and HMBP1 induce the release of pro-inflammatory cytokine and immune thrombosis, respectively (Al-kuraishy et al. 2022a). HMBP1 induction in COVID-19 aggravates the recruitment of neutrophils with the formation of neutrophil extracellular traps (NETs), which causes more inflammation and thrombosis termed immune thrombosis (Al-Kuraishy et al. 2022c, 2022m). Interestingly, Nrf2 agonist resveratrol suppresses inflammatory reactions and inhibits the expression of MCP-1, TNF-α, IL-6, and IL-1β, as well as the expression of adhesion molecules through activating the Nrf2/HO-1 pathway (Al-Kuraishy et al. 2022a; Giordo et al. 2021). Resveratrol may have potential therapeutic efficacy in mitigating SARS-CoV-2 infection-associated hemostatic complications and disorders (Giordo et al. 2021; Liao et al. 2021). Giordo et al. (Giordo et al. 2021) proposed that in virtue of its anti-thrombotic, antioxidant, and anti-inflammatory effects, resveratrol can reduce OS, inflammatory disorders, and thrombotic events in COVID-19 through activation of the Nrf2/HO-1 pathway (Giordo et al. 2021).

##### PI3K/Akt signaling

Certainly, activated PI3K/Akt signaling is necessary for the anti-inflammatory effect of Nrf2 (Al-Kuraishy et al. 2022w; Basile et al. 2022). However, PI3K/Akt pathway may enhance SARS-CoV-2 endocytosis mediated by the clathrin pathway (Khezri et al. 2022). Deregulation of renin-angiotensin system (RAS) in SARS-CoV-2 infection also provokes increasing of AngII, which is a potent activator of this pathway with the development of lung fibrosis (Al-Kuraishy et al. 2022r, 2022v, 2022x; Hussien et al. 2021). PI3K/Akt also activates the NF-κB pathway with further inflammatory and OS disorders in COVID-19 (Basile et al. 2022). Azithromycin suppresses PI3K/Akt pathway by inhibiting abnormal inflammatory reactions in COVID-19 (Al-Kuraishy et al. 2021e, 2020c, 2020d). Based on this evidence, increased activation of PI3K/Akt signaling encourages the expression of anti-inflammatory Nrf2 to neutralize hyperinflammation and OS in COVID-19.

##### Mechanistic target of rapamycin (mTOR)

Of note, there is a potential interaction between Nrf2 and mTOR, which is concerned in the propagation of inflammatory disorders (Al-Kuraishy et al. 2022e, 2022s). Sestrins which is triggered by environmental stressors promote the expression of Nrf2 and inhibit the mTOR pathway (Rhee and Bae 2015). In addition, Nrf2 blocks the activation of the mTOR pathway during inflammatory reactions (Rhee and Bae 2015). Besides, Nrf2 agonist sulforaphane reduces OS and normalizes autophagy in Parkinson’s disease through inhibition generation of ROS and mTOR pathways, respectively (Zhou et al. 2016). In COVID-19, the mTOR pathway promotes viral replication (Karam et al. 2021). Particularly, mTOR is a serine/threonine kinase that controls cell growth by enhancing mTOR1 and mTOR2 (Battaglioni et al. 2022). It has been identified that mTOR inhibitor metformin effectively treats influenza virus infection (Al-Kuraishy et al. 2021b, 2022i). It has been suggested that the mTOR pathway is essential for the replication of SARS-CoV-2, and mTOR inhibitors and modulators like sapanisertib and metformin, respectively, may reduce COVID-19 severity (Coleman et al. 2021). In this state, Nrf2 signaling may decrease the severity of SARS-CoV-2 infection and associated complications through modulation of the mTOR pathway.

##### Advanced glycation endproducts

Advanced glycation endproducts (AGEs) provoke the release of pro-inflammatory cytokines and induce the propagation of OS and inflammatory disorders with subsequent activation of the Nrf2 pathway to counterbalance OS and/or inflammatory reactions (Al-Kuraishy et al. 2023d, 2021i, 2020e; Alomair et al. 2022a). Remarkably, Nrf2/HO-1/NQO1 axis is exceedingly activated in the endothelial cells subjected to AGEs activators to give an adaptive response against OS and/or inflammatory reactions in diabetes (He et al. 2011). An experimental study established that some herbal medicine like *Eucommia ulmoides* attenuates glucotoxicity by inhibiting AGEs through enhancement of the Nrf2 pathway in diabetic mice (Do et al. 2018). These findings suggest that Nrf2 is considered as a potent inhibitor of AGEs in different metabolic and inflammatory disorders. AGEs are generated due to the glycation of DNA, proteins, and lipids, which play a critical role in the pathogenesis of metabolic and inflammatory disorders (Alomair et al. 2022a). The receptor of AGEs (RAGE) expressed in pulmonary epithelial alveolar cells is involved in the pathogenesis of SARS-CoV-2 infection and associated lung inflammation, ALI and ARDS (Al-Kuraishy et al. 2022b, 2021h; Alkazmi et al. 2022). Both diabetes and the aging process accelerate the production of AGEs which, via the interaction with RAGE on the macrophages, trigger lung inflammation in COVID-19 (Alomair et al. 2022b; Al-Kuraishy et al. 2022j). Furthermore, Nrf2 is reduced in different metabolic disorders, including diabetes mellitus (Costa et al. 2019). This may explain the susceptibility of patients with metabolic disorders to the risk of SARS-CoV-2 infection and COVID-19 severity (Batiha et al. 2023b; Al-Kuraishy et al. 2021g, Al-Kuraishy et al. 2023f). Likewise, soluble RAGE (sRAGE) plasma level is associated with COVID-19 severity. A prospective cohort study included 164 COVID-19 patients compared to 23 non-COVID-19 pneumonia demonstrated that high sRAGE plasma level was associated with the need for oxygen therapy and 30-day mortality (Lim et al. 2021). Consequently, sRAGE is a potential biomarker for predicting COVID-19 severity and mortality.

##### Signal transducer and activator of transcription 3

Signal transducer and activator of transcription factors (STATs) are a family of transcription factors that regulate cell growth, survival, differentiation, and motility (Diallo and Herrera 2022). STAT3 protein exists in a latent or inactive form in the cytoplasm (Diallo and Herrera 2022). STAT3 can be activated by receptor-associated kinases and phosphorylated at various phosphorylation sites, particularly at Tyr-705 and Ser-727 (Dai et al. 2022; Al-Kuraishy et al. 2023h). STAT3 protein is expressed at a basal level in cells but rapidly increases once activated by specific cytokines (Al-Kuraishy et al. 2022d). STAT3 is a critical factor in interleukin-6 (IL-6) induced gene regulation. STAT3 can be phosphorylated by IL-6 signal pathway, whereas IL-6 can also activate STAT3 at the transcriptional level (Al-Thomali et al. 2022b). STAT3 signaling pathway is exaggerated in SARS-CoV-2 infection leading to hyperinflammation, thrombosis, and lung fibrosis (Al-Kuraishy et al. 2022l). STAT3 impairs antiviral immune response and the development of lymphopenia (Al-Kuraishy et al. 2022d). Herein, targeting STAT3 in COVID-19 may mitigate hyperinflammation and related fatal complications (Batiha et al. 2022c). It has been revealed that the Nrf2 pathway negatively regulates STAT3 expression in rats with benign prostatic hypertrophy (Fishel et al. 2015). Downregulation of Nrf2 increases ferroptosis-induced ALI by activating STAT3 expression in mice (Wang et al. 2022). Therefore, the Nrf2 pathway plays a crucial role in attenuating inflammatory disorders in COVID-19 through inhibition of STAT3.

##### ADAM-metalloproteinase domain 17

ADAM-metalloproteinase domain 17 (ADAM17) is a ubiquitously expressed membrane-bound enzyme that mediates shedding of a wide variety of important regulators in inflammation including cytokines and adhesion molecules (Nadwa et al. 2023; Aleksova et al. 2021; Almishri et al. 2022; Al-Kuraishy and Al-Gareeb 2021a). ADAMs, similarly to MMPs, possess various physiological functions and the ability to regulate many processes such as cell migration, proliferation, angiogenesis, apoptosis, wound healing, and tissue repair and survival. ADAM17 activates TNF-α and sheds ACE2, facilitating SARS-CoV-2 entry (Nadwa et al. 2023; Aleksova et al. 2021; Almishri et al. 2022; Al-Kuraishy and Al-Gareeb 2021a). In spite of a lower ACE2 expression on cells surface, patients with cardiovascular disorders have a higher COVID-19 mortality rate, which is likely driven by the imbalance between ADAM17 protein which is required for cleavage of ACE2 ectodomain resulting in increased ACE2 shedding and TMPRSS2 which is required for spike glycoprotein priming (Aleksova et al. 2021; Almishri et al. 2022; Al-Kuraishy and Al-Gareeb 2021a). Although the membrane-bound form of ACE2 regulates the ACE2/Ang1-7 axis, the role of soluble ACE2 remains largely unclear (Al-Buhadily et al. 2021). It has been recognized that activating the Nrf2 pathway by butyrate releasers reduces COVID-19 severity by inhibiting the ADAM17 pathway (Paparo et al. 2022). Notably, the expression of ADAM17 was significantly increased in Nrf2-deficient macrophages in vivo and in vitro (Reddy et al. 2022). Therefore, promoting expression of Nrf2 reduces cardiovascular injury and inflammatory injury in COVID-19 patients.

These verdicts pointed out that the Nrf2 pathway is intricate with different signaling pathways to reduce the risk of OS and inflammatory disorders in COVID-19.

### Nrf2 and renin angiotensin system

SARS-CoV-2 infection induces downregulation of ACE2, which involves the metabolism of AngII to angiotensin 1–7 (Ang1-7) (Al-Kuraishy et al. 2022o, 2023j, 2023k). This interaction leads to the overexpression of pro-inflammatory AngII and the reduction of anti-inflammatory Ang1-7 with subsequent development of ALI/ARDS (Al-Kuraishy et al. 2023l, 2022k, 2022u, 2023g). Deregulation of the RAS is associated with the development of OS and inflammation by inducing the expression of NADPH and pro-inflammatory cytokines, respectively (Al-Kuraishy et al. 2021m). A higher circulating AngII level inhibits endogenous antioxidant capacity and may inhibit the expression of the Nrf2 pathway (Al-Kuraishy et al. 2021n; Alkazmi et al. 2023a). Exaggerated AngII in rats with experimental kidney injury leads to OS by inhibiting the Nrf2 pathway (Uddin et al. 2021). Pepe et al. (Pepe et al. 2019) observed that induction of the Nrf2 pathway attenuates AngII-induced intestinal epithelial injury in mice. In addition, activators of the Nrf2 pathway can reduce exaggerated intra-renal AngII in diabetic patients and the development of diabetic nephropathy (Abdo et al. 2015). The SARS-CoV-2 viral spike protein binds to ACE2, which aids viral entrance into the host cell (Al-Kuraishy et al. 2023c, 2023e, 2022b; Alsaidan et al. 2023). In light of the probable elevation the expression of ACE2 by these drugs, there has been growing suspicion that ACE inhibitors and Ang II receptor blockers may raise the risk of the onset and severity of COVID-19 (Al-Kuraishy et al. 2022h). SARS-CoV-2, on the other hand, stimulates ACE2 shedding from the cell surface, downregulates ACE2 expression, and enhances ACE2 endocytosis, which raises Ang II concentration and lowering Ang-(1–7) (Al-Maiahy et al. 2021). Due to Ang II’s pro-inflammatory effects and the lack of Ang-(1–7)-mediated counter-regulation is probably significant in the pathophysiology of COVID-19 (Al-Kuraishy and Al-Gareeb 2021c). COVID-19 hypercoagulability may be caused by the prothrombotic effects of increased Ang II (Al-Kuraishy et al. 2023b). The COVID-19-associated vasculopathy may be facilitated by the upregulation of ACE/Ang II and downregulation of ACE2/Ang-(1–7) in the vascular endothelium (Al-kuraishy et al. 2023b). An updated study revealed that local RAS contribute to the pathogenesis and progression of diabetic nephropathy by exacerbating oxidative stress and inflammation (Razliqi et al. 2023). Activation of Nrf2 pathway by gentisic acid attenuates diabetic nephropathy in animal model study (Razliqi et al. 2023). Therefore, dysregulation of RAS in COVID-19 could be the possible reason behind the reduction of Nrf2 activity. Thus, AT1R blockers may be beneficial in preventing OS and inflammatory reactions via upregulation of the Nrf2 pathway (Karan et al. 2020).

### Nrf2 and endothelial dysfunction

SARS-CoV-2 infection primarily affects the vascular endothelium leading to endothelial dysfunction and the development of coagulopathy (Alrouji et al. 2023b; Alkazmi et al. 2023b; Alomair et al. 2023). SARS-CoV-2 infects the endothelial cells directly due to the abundance expression of ACE2, causing cellular injury and apoptosis with subsequent reduction of endothelial cells’ capacity to release anti-thrombotic factors (Alomair et al. 2023). In addition, injury of pulmonary vascular endothelial cells by direct cytopathic effects of SARS-CoV-2 or due to OS and hyperinflammation lead to pulmonary micro-thrombosis, a prominent feature of COVID-19 (Moubarak et al. 2021; Batiha et al. 2023b; Al-Kuraishy and Al-Gareeb 2021b). Particularly, circulating endothelial cells and soluble intercellular adhesion molecule-1 levels are augmented in severely affected COVID-19 patients (Bonaventura et al. 2021). ED is a risk factor for developing micro-vascular dysfunction and immune thrombosis due to NETs formation and platelet activation (Bonaventura et al. 2021). It has been reported that activation of the Nrf2 pathway by ellagic acid prevents OS-induced ED in mice (Ding et al. 2014). Chen et al. (Chen et al. 2015) illustrated that Nrf2 activators decrease the propagation of ED in animal model studies. It has been demonstrated that resveratrol has an essential role in preventing the development of ED through the activation of the Nrf2 pathway (Parsamanesh et al. 2021). In addition, Nrf2 improves endothelial function by activating nitric oxide (NO) synthase and the release of NO (Luo et al. 2015). Furthermore, Nrf2 tempers the development of immune thrombosis and coagulopathy through the mitigation of hyperinflammation and OS, which are intricate in the propagation of ED and linked coagulopathy (Takahashi et al. 2020). In vitro study conducted by Takahashi et al. (Takahashi et al. 2020) demonstrated that Nrf2 plays an essential role in the prevention of coagulopathy by negative regulation of tissue plasminogen activator and fibrinolytic activity. As well, activation of Nrf2 reduces the risk of venous thrombosis by alleviating inflammatory changes and OS (Li et al. 2021). These verdicts suggested that the Nrf2 pathway may mitigate ED and associated coagulopathy in COVID-19.

### Nrf2 and cytokine storm in COVID-19

SARS-CoV-2 can rapidly activate pathogenic Th1 cells to secrete pro-inflammatory cytokines, such as granulocyte–macrophage colony-stimulating factor (GM-CSF) and IL-6 (Al-Kuraishy et al. 2021d, 2021f, 2020b; Onohuean et al. 2021). GM-CSF further activates inflammatory monocytes to produce large quantities of IL-6, TNF-α, and other cytokines (Al-Kuraishy et al. 2020a). Membrane-bound immune receptors such as TLR4 may contribute to an imbalanced inflammatory response, and weak IFN-γ induction may be an important amplifier of cytokine production (Hussien et al. 2018). Together, the impaired acquired immune responses and unrestrained inflammatory innate responses to SARS-CoV-2 may cause cytokine storms (Rasheed et al. 2019; Song et al. 2020). It has been shown that the Nrf2 pathway inhibits the expression of pro-inflammatory cytokines and the progression of cytokine storm in COVID-19 (Zinovkin and Grebenchikov 2020). Particularly, Nrf2 has a role in regulating immune response and inflammation. It decreases the expression of pro-inflammatory cytokines, including MCP-1, TNF-α, IL-6, and IL-1β, by inhibiting the recruitment of RNA polymerase II and macrophage activation (Zhang et al. 2022). In critically affected COVID-19, Nrf2 activators decrease systemic inflammation and OS (McCord et al. 2020). Different experimental studies confirmed that Nrf2 activators inhibit the expression and release of pro-inflammatory cytokines (Motterlini et al. 2019; Thimmulappa et al. 2006). Similarly, Nrf2 activators attenuate the airway inflammatory process and ED development (Al-Kuraishy et al. 2019a, 2019b, 2022k). A clinical trial also revealed the protective effect of Nrf2 activators in preventing lung inflammation (Kobayashi et al. 2016). Furthermore, Nrf2 inhibits the activation of different inflammatory signaling pathways, including NLRP3 inflammasome, TLR4, HMBP1, NF-κB, and STAT3 that are involved in the development of cytokine storm (Ren et al. 2019). As well, Nrf2 blocks the OS pathway, which activates inflammatory signaling pathways like NF-κB and NLRP3 inflammasome (Ren et al. 2019). Similarly, Nrf2 inhibits abnormal and exaggerated immune response through inhibition of INF activation, thereby preventing the excessive release of pro-inflammatory cytokines (Bhaskar et al. 2020). Therefore, the Nrf2 activator could be effective in the attenuation of the SARS-CoV-2 infection-induced cytokine storm.

### Nrf2 activators and KEAP1 inhibitors

Sources and mechanism of actions of Nrf2 activators are listed (Table 1). It has been reported that Nrf2 activators reduces airway inflammation as documented by many clinical trials (Al-Kuraishy et al. 2022o; Carlson et al. 2020; Müller et al. 2016). For example, a flavonoid sulforaphane inhibits SARS-CoV-2 infection-induced expression of IL-6 and IL-8 in bronchial epithelial cells (Gasparello et al. 2021). Sulforaphane hinders the interaction between SARS-CoV-2 spike protein and ACE2 with inhibition of the release of pro-inflammatory cytokines and development of cytokine storm (Gasparello et al. 2021). Kiser et al. (Kiser et al. 2021) revealed that sulforaphane inhibits the expression of NLRP3 inflammasome and NF-κB with a suppression effect on the development of cytokine storms. Therefore, sulforaphane could be a candidate for treating COVID-19 through modulation of OS and hyperinflammation.

**Table 1:** Sources and mechanism of actions of Nrf2 activators

Ref	Nrf2 activator	Source	Mechanisms
Yagishita, Fahey (Yagishita et al. 2019)	Sulforaphane	Broccoli	Increases NQO1, inhibits OS, reduces misfolded proteins
Alam, Ali (Alam et al. 2022)	Epigallocatechin	Green tea	Increases antioxidant capacity
Wang, Wang (Wang et al. 2018)	Resveratrol	Grapes	Increases antioxidant capacity. A negative regulator of KEAP1 increases NQO1 expression
Jia, Zhang (Jia et al. 2021)	Quercetin	Apples, citrus fruits	Augmentation translocation of nuclear Nrf2
Cao, Zhao (Cao et al. 2021)	Lycopene	Watermelon, grapefruits	Improves HO-1 signaling, reduces ROS, inhibits apoptosis
Khalil, Eliwa (Khalil et al. 2021)	Triterpene lactones	Ashwagandha	Augmentation translocation of nuclear Nrf2 inhibits apoptosis
Korenori, Tanigawa (Korenori et al. 2013)	Isothiocyanate	Wasabi	Inhibits degradation of Nrf2
He, Li (He et al. 2020)	Thymol	Thyme	Upregulates Nrf2expression and inhibits KEAP1
Mimura, Inose-Maruyama (Mimura et al. 2019)	Carnosic acid	Rosemary	Inhibits KEAP1
Han, Xiao (Han et al. 2016)	Dimethyl fumarate	…………	Inhibits ROS generation activates ARE
Macabrey, Longchamp (Macabrey et al. 2022)	Sodium thiosulfate	………….	Inhibits degradation of Nrf2 and ROS generation

Moreover, dimethyl fumarate, an approved drug for treating multiple sclerosis and psoriasis, inhibits inflammatory disorders in both Nrf2-dependent and independent pathways (Al-Kuraishy et al. 2023i). A case study of COVID-19 patients treated with dimethyl fumarate demonstrated that this drug had immunomodulatory effects that can prevent the development of cytokine storm (Mantero et al. 2021). Furthermore, dimethyl fumarate had antioxidant and anti-inflammatory effects with modulatory effects on the immune cells so that it can attenuate the development of cytokine storms (Timpani and Rybalka 2020). Dimethyl fumarate inhibits neutrophil migration, neutrophil-mediated ROS production, and pro-inflammatory cytokine expression (Müller et al. 2016). Remarkably, dimethyl fumarate competes with SARS-CoV-2 to bind nicotinic acetylcholine receptor, which is involved in the pathogenesis of SARS-CoV-2 infection and the development of dysautonomia (Simões et al. 2021). Therefore, dimethyl fumarate could effectively reduce the development of COVID-19-induced dysautonomia. Likewise, a hydrogen sulfide donor sodium thiosulfate induces activation of Nrf2 and used in the management of cyanide intoxications, has antiviral and anti-inflammatory properties, and could be effective against SARS-CoV-2 infection (Dai et al. 2021). Importantly, sodium thiosulfate inhibits OS by reducing the production of ROS. Sodium thiosulfate has a cytoprotective effect by inhibiting pro-inflammatory cytokines (Marutani et al. 2015). Sodium thiosulfate prevents pneumonia-induced ALI in children (Farese et al. 2011). Therefore, inhalation of sodium thiosulfate could efficiently decrease SARS-CoV-2 infection-induced ALI (Evgen’ev and Frenkel 2020).

Indeed, resveratrol, a plant polyphenol, activates Nrf2 and inhibits the KEAP1 pathway, improving the anti-inflammatory and antioxidant properties of the Nrf2 signaling pathway (Liao et al. 2021). Similarly, resveratrol augments endogenous antioxidant capacity independent of the Nrf2 signaling pathway (Liao et al. 2021). Consequently, resveratrol can be used as adjuvant therapy in managing COVID-19 patients through the alleviation of OS and inflammatory disorders (Russo et al. 2023). A study demonstrated that a diterpenoid lactone and rographolide inhibits the interaction between Nrf2 and KEAP1, leading to the upregulation of Nrf2 expression (Schulte et al. 2022). Therefore, rographolide could be effective against SARS-CoV-2 infection-induced OS.

These findings proposed that activation of Nrf2 by direct activators or inhibition of the KEAP1 pathway augment the anti-inflammatory and antioxidant effect of the Nrf2 pathway. In this state, activation of the Nrf2 pathway can attenuate the development of OS, hyperinflammation, and cytokine storm.

On the other hand, KEAP1 inhibitors increase the activity of anti-inflammatory and antioxidant effects mediated by Nrf2. KEAP1 inhibitors attenuate inflammatory and OS-mediated ALI (Duran et al. 2016). KEAP1 inhibitors like pentoxifylline and pirfenidone improve the antioxidant capacity and reduce COVID-19 severity in patients with ALI/ARDS (Chavarría et al. 2021; Hamidi et al. 2021). In addition, KEAP1 inhibitors prevent the activation of NF-κB and the release of pro-inflammatory cytokines (Bhandari et al. 2021).

Quercetin, a well-known antioxidant was studied in 152 outpatients suffering from COVID-19 (Singh et al. 2021). The randomized controlled and open-labeled study was carried out for 30 days to show that quercetin is helpful as an adjuvant to the standard treatment in COVID-19 patients (Singh et al. 2021). It was reported that during the initial stage of COVID-19 infection, quertcetin reduced the duration of hospitalization, the need for oxygen supplementation and deaths (Pierro et al. 2021). Quercetin activates Keap1-Nrf2 system and has been reported to mediate anti-inflammatory response (Qin and Hou 2016).

Therefore, Nrf2 activators and KEAP1 inhibitors may alleviate OS and inflammatory changes and prevent cytokine storm development in COVID-19. Indeed, there are a lot of Nrf2 activators, but most of them are not approved by FDA. Nevertheless, because of their anti-inflammatory and antioxidant properties, Nrf2 activators could be a possible adjuvant therapeutic strategy in managing severely affected COVID-19 patients.

## Limitation

The present critical review had several limitations, including a shortage of clinical studies which intricate Nrf2 activators in the management of COVID-19 patients. Likewise, most clinical trials still do not endorse Nrf2 activators in the management of COVID-19. Targeting Nrf2 by modulators might be helpful in COVID-19. Though, before implementing this novel strategy in this current pandemic, we must address a number of important issues, including a clear concept of SARS-CoV-2–2 interactions, other impacts of downregulation of ACE2 in human lung, clear concepts on the metabolic reprogramming, and adaptation of immune cells such as macrophages and T cells, pharmacological activation of Nrf2and its impact on the viral entry into the host cell. Taken together, more research is necessary with adequate preclinical and clinical trials to establish this strategy.

## Conclusions

SARS-CoV-2-induced OS triggers the activation of different signaling pathways, which counterbalances this complication. One of these pathways is Nrf2 which induces a series of cellular interactions to mitigate SARS-CoV-2-mediated viral toxicity and OS-induced cellular injury. The nrf2 pathway inhibits the expression of pro-inflammatory cytokines and the development of cytokine storm in COVID-19. Nrf2 activators could play an essential role in reducing SARS-CoV-2 infection-induced inflammation by suppressing NLRP3 inflammasome in COVID-19. Furthermore, Nrf2 activators can attenuate ED, RAS dysregulation, immune thrombosis, and coagulopathy. Therefore, this review suggests experimental, in vitro, preclinical, and clinical studies to confirm the possible therapeutic effects of Nrf2 activators alone or as adjuvant therapy in the management of COVID-19.

## Data Availability

Not applicable.
